# Left common carotid artery arising from the brachiocephalic trunk: a case report

**DOI:** 10.1186/1757-1626-1-83

**Published:** 2008-08-11

**Authors:** Georgios Paraskevas, Paris Agios, Marios Stavrakas, Alexandra Stoltidou, Alexandros Tzaveas

**Affiliations:** 1 Department of Anatomy, Medical Faculty of Aristotle University of Thessaloniki, P.O. Box: 300, P. Code: 54124, Thessaloniki, Greece

## Abstract

An abnormal origin of the left common carotid artery from the initial portion of the brachiocephalic trunk was found in the superior mediastinum in a 81-year-old Caucasian male cadaver during dissection practice. We report on the exact morphology of that variant that is appeared in an incidence of 0,2% in the literature. We discuss the relative literature and pay attention on the significance of such a variation for clinicians in its recognition and protection.

## Introduction

Increasing activity in the fields of cardiac and vascular surgery has served to revive interest in the developmental and adult anatomy of the aortic arches and the great vessels derived therefrom. In the course of study of specimens in the Laboratory of Gross Anatomy, it became abundantly and strictly evident that the "standard" type of branching from the aortic arch not only existed in the preponderant number of cases, but also, when placed with a rather ordinary variation thereof, gave a combined total that represented over 90 per cent of cases in a series of 1000 specimens [1].

The summit of the arch is usually 2,5 cm approximately below the superior sternal border but may diverge from this. Sometimes the aorta curves over the right pulmonary hilum (as a right aortic arch) descending to the right of the vertebral column, accompanied by a transposition of thoracic and abdominal viscera. Less often, after arching over the right hilum, it passes behind the oesophagus to its usual position; this is not accompanied by visceral transposition. The aorta may divide into ascending and descending trunks, sometimes dividing near its origin and the two branches soon reuniting; the oesophagus and trachea usually pass through the interval between them [2].

As far as the branches of the aortic arch are concerned, there is a plenty of variations in the origins of them. An analysis of variation in branches from 1000 aortic arches showed the following findings: In 27%, the left common carotid artery originates from the brachiocephalic trunk. In 2,5%, each of the four arteries originate independently from the arch of the aorta, while in 1,2%, right and left brachiocephalic trunks originate from the arch of the aorta. The most common pattern in 65% is formed by the separate origination of three branches springing from the vessel's convex aspect: the brachiocephalic trunk, left common carotid and left subclavian arteries [3]. In our work we present a rare type of left common carotid artery origin from the initial portion of the brachiocephalic trunk.

## Case presentation

**Figure 1:**
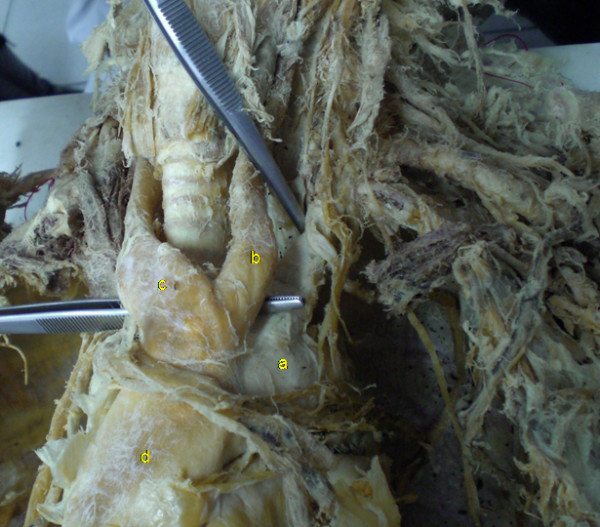
**Picture of the cadaveric preparation.** The left common carotid artery (b) is shown arising from the initial portion of the brachiocephalic trunk (c) (a: aortic arch, d: ascending aorta).

We dissected a 31-year-old, Caucasian, male, formaline-fixed cadaver. His ethnicity was Greek. His weight was 83 kg and the height 1,78 m. He had no past medical history and was on no medication. He did not use to smoke or drink (according to his next of kin). The dissection was carried out as part of the practice for the medical students and was approved by the Ethical Committee of the University. During the anatomical preparation we came across a variation referring to the branches springing from the aortic arch. After resection of the anterior thoracic wall we removed carefully the fat tissue and the pericardium covering the ascending aorta and the great vessels arising from it. Having obtained a clear view of the great vessels we noticed the presence of a left common carotid artery arising from the left surface of the origin site of the brachiocephalic trunk (Figure 1).

## Discussion

Three branches, as it is known, spring from aortic arch convex aspect: the brachiocephalic trunk, left common carotid and left subclavian arteries [4]. The ascending aorta arises from the base of the left ventricle behind the left sternal margin opposite the third costal cartilage. The arch of the aorta lies behind the lower part of the sternal manubrium. It begins behind the right border of the sternum at the level of the second rib cartilage, and extends dorsally to the left to reach the spine at the left of the body of the fourth thoracic vertebra [2]. The excision of the right lobe of the thyroid gland reveals the relation of the carotid and the subclavian arteries and the intervening portion of the aortic arch to the trachea [1]. They may branch from the beginning of the arch of the upper part of the ascending aorta; the distance between these origins varies, the most frequent being approximation of the left common carotid artery to the brachiocephalic trunk [2]. Primary branches may be reduced to one, more commonly two, the left common carotid arising from the brachiocephalic trunk (7%) [2], while Anson [3] rise this incidence to 27%. However Anson [3] referred to the presence of a left common carotid artery arising from the initial portion of the brachiocephalic trunk in a frequency of 0,2%. Because of the many changes involved in transformation of the embryonic aortic arch system into the adult arterial pattern, it is understandable that variations may occur. Most anomalies result from the persistence of parts of the aortic arches that normally disappear or from disappearance of parts that normally persist. Several kinds of uncommon defect occur when arches persist instead of becoming obliterated or vice versa. A right aortic arch occurs when the left fourth arch and dorsal aorta disappear. If the left fourth arch alone (and not the dorsal aorta as well) disappears, the condition of interrupted arch arises; the first part of the arch gives off the brachiocephalic and left common carotid vessels, and beyond the gap the pulmonary trunk and a persistent patent ductus (sixth arch) are required to complete an "arch" with the left dorsal aorta. If the right dorsal aorta persists as well as the left, a double arch ensues with the trachea and oesophagus clasped between the two [4,5]. Of course the analysis of aortic arch variability in morphology is beyond the aim of our study.

In Anson's analysis of variation in branches from 1000 aortic arches there was a 65% of the usual pattern, a 25% of the four large arteries branching separately, the remaining 5% showed a great variety of patterns, the commonest (1,2%) being symmetrical right and left brachiocephalic trunks [3].

More rarely, the left common carotid and subclavian arteries arise from a left brachiocephalic or right common carotid and subclavian arteries arise separately, in which case the latter more often branches from the left end of the arch and passes behind the oesophagus [1,2,6,7]. This anomaly assumes some importance in the adult as well as in the child, as a cause of esophageal compression. The abnormal course of the "recurrent" laryngeal nerve, which accompanies this anomaly, is also important [1].

The left vertebral artery may arise between the left common carotid and the subclavian. Very rarely, external and internal carotid arteries arise separately, the common carotid being absent on one or both sides, or both carotids and one or both vertebrals may be separate branches. In about 12% the right common carotid artery arises above the level of the sternoclavicular joint or it may be a separate branch from the aorta; again it may arise with its fellow. The left common carotid artery varies in origin more than the right.

When a "right aorta" occurs, the arrangement of its three branches is reversed. The common carotids may have a single trunk, the subclavians separate, the right arising from the left end of the arch. Other arteries may branch from it, most commonly one or both bronchial arteries and the arteria thyroide ima [2].

As it is known specific interest is shown in surgery with respect to the relation of an anomalous arch or arches to the viscera in the neck and the thorax. Additionally, a variant of origin and course of a great vessel arising from the aortic arch is of great clinical value, because the ignorance on behalf the surgeon of such a variation may cause serious surgical complications during procedures occurring in the superior mediastinum and the bare of neck.

## Competing interests

The authors declare that they have no competing interests.

## Authors' contributions

GP did the dissection and supervised the manuscript writing. AP performed the literature review and obtained the written consent. MS and AS obtained the photos and wrote the draft of the manuscript. AT helped to the final writing of the paper. All authors read and approved the final manuscript.

## Consent

A written consent was obtained by the cadaver's next of kin for publication of the article.

## References

[B1] McVay CB (1984). Anson and McVay Surgical Anatomy.

[B2] Williams PL, Warwick R, Dyson M, Bannister LH (1989). Gray's Anatomy.

[B3] Anson BH (1963). The aortic arch and its branches. Cardiology.

[B4] McMinn R (1990). Last's Anatomy Regional and Applied.

[B5] Moore KL, Persaud TV (1993). The Developing Human, Clinically Oriented Embryology.

[B6] Agur A (1991). Grant's Atlas of Anatomy.

[B7] Healey J, Hodge J (1990). Surgical Anatomy.

